# Protective Effects of PARP-1 Knockout on Dyslipidemia-Induced Autonomic and Vascular Dysfunction in ApoE^−/−^ Mice: Effects on eNOS and Oxidative Stress

**DOI:** 10.1371/journal.pone.0007430

**Published:** 2009-10-13

**Authors:** Chetan P. Hans, Yumei Feng, Amarjit S. Naura, Mourad Zerfaoui, Bashir M. Rezk, Huijing Xia, Alan D. Kaye, Khalid Matrougui, Eric Lazartigues, A. Hamid Boulares

**Affiliations:** 1 Department of Pharmacology and Experimental Therapeutics, Louisiana State University Health Sciences Center, New Orleans, Louisiana, United States of America; 2 Department of Anesthesiology Louisiana State University Health Sciences Center, New Orleans, Louisiana, United States of America; 3 Department of Physiology, Tulane University Medical Center, New Orleans, Louisiana, United States of America; National Institutes of Health (NIH) / National Institute of Environmental Health Sciences (NIEHS), United States of America

## Abstract

The aims of this study were to investigate the role of poly(ADP-ribose) polymerase (PARP)-1 in dyslipidemia-associated vascular dysfunction as well as autonomic nervous system dysregulation. Apolipoprotein (ApoE)**^−/−^** mice fed a high-fat diet were used as a model of atherosclerosis. Vascular and autonomic functions were measured in conscious mice using telemetry. The study revealed that PARP-1 plays an important role in dyslipidemia-associated vascular and autonomic dysfunction. Inhibition of this enzyme by gene knockout partially restored baroreflex sensitivity in ApoE^−/−^ mice without affecting baseline heart-rate and arterial pressure, and also improved heart-rate responses following selective blockade of the autonomic nervous system. The protective effect of PARP-1 gene deletion against dyslipidemia-induced endothelial dysfunction was associated with preservation of eNOS activity. Dyslipidemia induced PARP-1 activation was accompanied by oxidative tissue damage, as evidenced by increased expression of iNOS and subsequent protein nitration. PARP-1 gene deletion reversed these effects, suggesting that PARP-1 may contribute to vascular and autonomic pathologies by promoting oxidative tissue injury. Further, inhibition of this oxidative damage may account for protective effects of PARP-1 gene deletion on vascular and autonomic functions. This study demonstrates that PARP-1 participates in dyslipidemia-mediated dysregulation of the autonomic nervous system and that PARP-1 gene deletion normalizes autonomic and vascular dysfunctions. Maintenance of eNOS activity may be associated with the protective effect of PARP-1 gene deletion against dyslipidemia-induced endothelial dysfunction.

## Introduction

Atherosclerosis, a major contributor to morbidity and mortality in developed countries, is the underlying cause of a number of cardiovascular diseases and is closely associated with dyslipidemia [1], [2]. Significant research has demonstrated that lipid-associated disorders, such as atherosclerosis, are linked to alterations in hemodynamic parameters, which may result in pathological cardiovascular events [1], [2], [3]. Hypertension and dyslipidemia are mechanistically linked and may act in synergy at the arterial wall to enhance atherogenesis. The autonomic nervous system serves as the main regulator of blood pressure and heart rate homeostasis, in part, by modulation of the arterial baroreflex [4]. Indeed, reduced spontaneous baroreflex sensitivity (SBRS) is associated with impaired cardiac autonomic balance in hypertension, coronary artery disease, and myocardial infarction [5], [6]. In addition, disruption in the balance between parasympathetic and sympathetic tones can lead to cardiovascular dysfunction [6], [7].

Endothelial dysfunction has been shown to be a reliable early marker for atherosclerosis [8]. Nitric oxide (NO), a product of endothelial NO synthase (eNOS), is released by the vascular endothelium in response to various stimuli including acethylcholine (Ach), and plays an important role in endothelium-dependent vasodilation. Molecular mechanisms responsible for endothelial dysfunction may include decreased expression of eNOS protein, alterations in the membrane signaling pathway leading to eNOS enzymatic activation (e.g., signaling-induced eNOS phosphorylation), and decreased NO bioavailability through oxidants such as superoxide (O_2_·^−^) [9], [10]. Numerous studies have demonstrated that hypercholesterolemia is associated with defects in NO-dependent endothelial function in humans and in many experimental models, including ApoE^−/−^ mice [11], [12]. Impaired endothelium-dependent relaxation in response to ACh occurs in the aorta [13] and the coronary arteries [14] of genetically-altered hyperlipidemic mice.

Our laboratory recently demonstrated that poly-ADP-ribose polymerase (PARP)-1 is activated within atherosclerotic plaques in an animal model of atherosclerosis [15]. Indeed, decreasing PARP-1 activity not only led to plaque stability, but actually promoted regression of pre-established atherosclerotic plaques [15], [16], [17]. These anti-atherogenic effects were associated with a reduction in inflammatory factors such as TNF, ICAM, monocyte chemoattractant protein (MCP-1). Additional effects include cellular changes related to plaque dynamics such as an increase in smooth muscle cell (SMC) content, decreased collagen degradation, and increased TIMP-2 expression [15], [17]. Support for these observations is provided by a recent report by von Lukowicz et et al [18].

Previous studies have demonstrated that PARP-1 activation might be involved in vascular dysfunction associated with circulatory shock, heart failure, ischemia reperfusion injury, hypertension, and diabetes [19], [20], [21], [22]. Inhibition of PARP-1 confers protection against many of these conditions [19], [20], [23], [24]. However, the exact mechanism by which PARP-1 mediates development of these diseases needs to be better clarified in order for PARP-1 inhibition to be a potential target as a viable therapeutic strategy for cardiovascular disorders.

In the present study, we used an integrative approach to assess the role of PARP-1 in the pathogenesis of atherosclerosis-associated vascular dysfunction and to investigate the effect of PARP-1 gene knockout on autonomic function and endothelium dysfunction. We also examined the relationship between occurrence of oxidative stress in our experimental model and PARP-1 activation as a potential mechanism for the manifestation of dyslipidemia-associated vascular and autonomic nervous system dysfunction.

## Materials and Methods

### Generation of PARP-1 and Double Knockout (DKO) Mice on C57BL/6 Background

C57BL/6 wild-type, ApoE^−/−^ (Jackson Laboratories, Bar Harbor, ME, USA), and PARP-1^−/−^ mice were housed and bred in a pathogen-free animal care facility at LSUHSC (New Orleans, LA) and allowed full access to standard mouse chow and water. All experimental protocols were approved by the LSUHSC Animal Care and Use Committee. C57BL/6 PARP-1^−/−^ mice were generated as described[25]. DKO mice were generated by crossing PARP-1 heterozygous mice with ApoE^−/−^ mice, as described [15]. All animals were genotyped using polymerase chain reaction (PCR). *ApoE^−/−^ mice fed a HF diet were used as a disease model for the present studies because of the pathogenesis of autonomic and vascular dysfunctions in these mice mimics that found in humans on a very short time-scale* [*11*] *.*

### Hemodynamic Parameters

*4–6 weeks old male ApoE^−/−^ and DKO mice (n = 6) were fed regular diet (RD) or high-fat diet (HF; Harlan Teklad, Madison, Wis) containing 21% fat by weight (0.2% cholesterol) for 16 weeks for histological studies. The fat contents of the diet are manipulated to induce changes that are linked to risk of cardiovascular disease in humans.* Separate male ApoE^−/−^ and DKO mice (n = 6) were fed under same diet regimen and anesthetized and implanted with a radiotelemetry probe as described[26]. Upon recovery from surgery (∼1 wk), blood pressure (BP) was recorded during 48 hours to determine baseline hemodynamic parameters (i.e. systolic and diastolic BP, mean arterial pressure (MAP) and heart rate (HR)). From baseline recordings, spontaneous baroreceptor reflex sensitivity (SBRS) was calculated using the sequence method as described[27]. Briefly, from previous BP and HR recordings, all up (increase in BP and decrease in HR) and down (decrease in BP and increase in HR) baroreflex sequences were detected using Hemolab software (http://www.harald.nxserve.net/HemoLab/HemoLab.php) and the average SBRS was calculated.

*Autonomic function* was assessed using a pharmacological method involving intraperitoneal injection (i.p.) of a β-blocker (propranolol, 8 mg/kg), a muscarinic receptor blocker (atropine, 1 mg/kg), or a ganglionic blocker (hexamethonium, 20 mg/kg). All drugs and doses were chosen for their ability to effectively block the respective receptors. Additionally, vascular function was assessed with acetylcholine (ACh; 2 mg/kg) and sodium nitroprusside (1 mg/kg; Sigma Chemicals). Changes in HR were analyzed 30 min after administration of the antagonists as described[28].

### Lipid profile, Histology, Immunohistochemistry, and Immunoblot Analysis

For lipid profile, animals were fasted for 4 h and samples were sent blindly to Cardiovascular Specialty Laboratories, Inc (Atlanta, GA) for lipid assessment. Perfusion fixed aortas from various genotypes were dissected and prepared for either Oil-Red-O staining using standard protocol or paraffin embedding. Tissues were sectioned (5 µm), subjected to hematoxylin and eosin (H&E) or trichrome staining. Lesion areas were assessed as described [15]. Hearts were paraffin embedded and sections were subjected to immunohistochemistry (IHC) using standard protocols[15], [16] and processed for eNOS (Cell Signal, Danvers, MA), phosphorylated eNOS (Sigma, St. Louis, MO), nitrotyrosine (Upstate, Lake Placid, NY), or iNOS (BD Bioscience, San Jose, CA) detection. Immunoblot analysis of protein extracts prepared from left ventricle of the different experimental groups was conducted as previously described [29]. Antibodies to eNOS or its phosphorylated form were used to probe nitrocellulose membranes.

### Real time PCR

RNA was extracted from left ventricle using the RNeasy Fibrous Tissue Mini Kit (Qiagen, Valencia, CA). Primers used for eNOS were as follows: forward, CAACGCTACCACGAGGACA; reverse: CTCCTGCAAAGAAAAGCTCTG from Sigma Genosys. (St. Louis, MO). The β−actin primers were described[15]. Amplification, detection and data analysis were performed using iCycler real-time PCR system (Bio-Rad Laboratories, Hercules, CA, USA).

### Statistical Analysis

Results are expressed as mean±SEM, from at least six mice per group unless stated otherwise. Two way or one-way ANOVA for repeated measures, followed by post hoc test, were performed when appropriate using GraphPad Prism 5 (GraphPad Software, San Diego, CA, USA). *Immunohistochemistry data represent arbitrary density units calculated using Pro-Image software and were summarized as a percent of basal control. For the comparison between the same groups on different diet regimen, one way ANOVA was employed to calculate statistical differences.* Differences were considered significant at *P*<0.05.

## Results

### PARP-1 Gene Deletion Reduces Atherosclerotic Lesion Formation in High Fat Diet-Fed C57Bl/6 ApoE^−/−^ Mice

**Figure 1:**
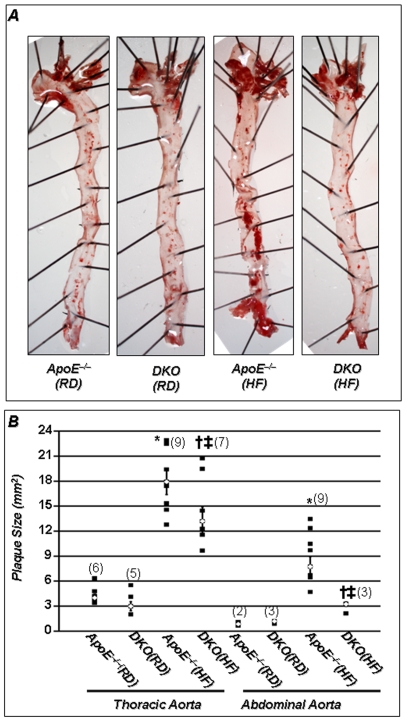
PARP-1 gene deletion promotes atherosclerotic plaque reduction in an animal model of atherosclerosis. (A) Aortas from the different experimental groups were formalin (PBS)-perfused and atherosclerotic plaques were visualized by *en face* Oil-Red-O staining. (B) Quantitation of plaque size and number (in parentheses) were determined as described[15]; *P* value (<0.05) *compared to ApoE^−/−^ RD, †compared to ApoE^−/−^ HF (16 weeks), and ‡compared to DKO mice on RD.

In order to verify that PARP-1 gene deletion protects against plaque formation in a murine model of atherosclerosis, aortas were collected from ApoE^−/−^ or DKO mice after a 16-week regimen with regular (RD) or high-fat (HF) diet. As expected, *en-face* oil red ‘O’ staining of longitudinally cut aortas revealed that HF diet induces numerous atherosclerotic plaques not only at the brachiocephalic or curvature areas of the thoracic region, but also all over the abdominal aorta (Figure 1A). PARP-1 gene deletion markedly lowered the incidence of these lesions along the aorta in HF fed mice, but this decrease was most prominent in the abdominal region (Figure 1A, B). Moreover, PARP-1 gene deletion not only significantly decreased plaque size but also plaque number (in parentheses) in ApoE^−/−^ mice fed HF diet for 16 weeks. It is important to note that ApoE^−/−^ mice fed RD for 16 weeks, developed few fatty streaks that were comparable to those observed in their DKO counterparts (Figure 1A). These results are consistent with previous observations from our laboratory using pharmacological inhibitor or heterozygous mice for PARP-1 on C57BL/6 genetic background[15] and findings from von Lukowicz et al [18] using PARP-1^−/−^ mice on a mixed genetic background.

### Lack of PARP-1 Improves Baroreflex Sensitivity without Affecting Baseline Hemodynamic Parameters

**Figure 2:**
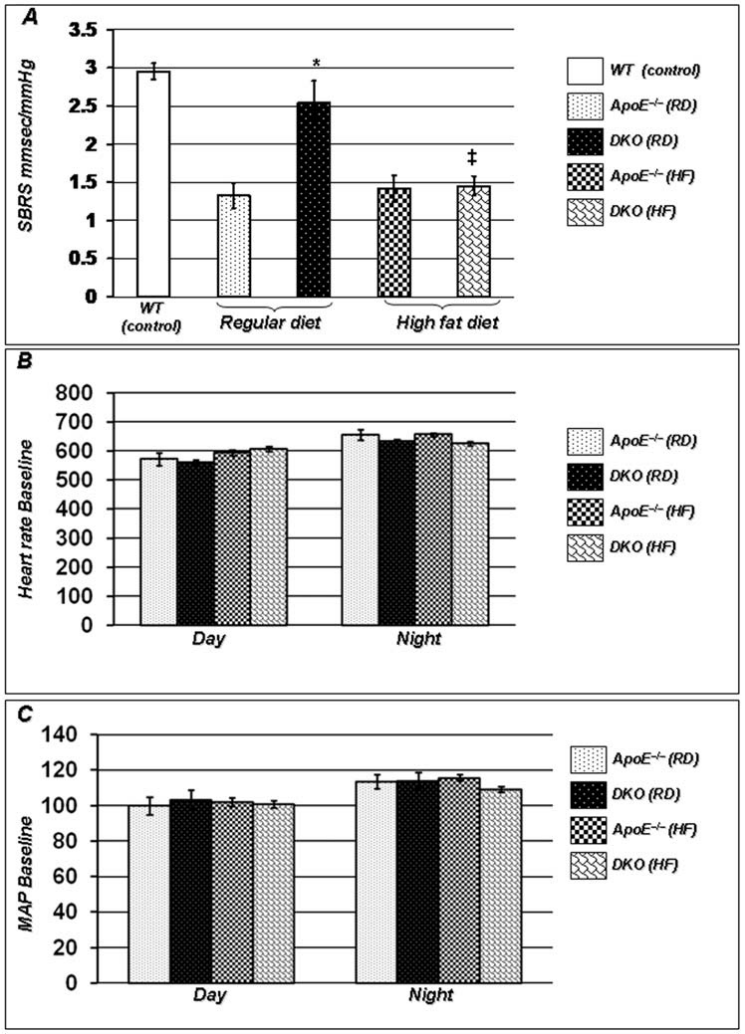
PARP-1 gene deletion improves spontaneous baroreflex sensitivity(SBRS). Data from telemetry recordings (average of 3 consecutive days) show restoration of SBRS by PARP-1 gene deletion in mouse model of atherosclerosis (A). Imroved SBRS response was not associated with significant difference in average HR (B) or MAP (C) during day or night among these groups. This effect was however abolished after feeding HF diet for 16 weeks (A). The data is expressed as mean ± SEM from at least 6 mice in each group for 3 different set of experiments. *P* value (<0.05) *compared to ApoE^−/−^ RD and ‡compared to DKO mice on RD.

One week prior to the completion of the diet regimens, the different experimental groups were implanted with telemetry probes for BP recording. Mice were then allowed to recover for one week before measurements. As shown in Figure 2A, ApoE^−/−^ mice on RD show a significant reduction of SBRS consistent with our previous observations[30]. PARP-1 gene deletion restored baroreflex sensitivity in these mice. This reversal was completely absent in HF-diet treated mice. Baseline heart rate (HR) and mean arterial pressure (MAP) did not significantly differ between groups and this was verified in the different phases of the nycthemere (Figure 2B and C). Overall, these results suggest that PARP-1 gene deletion may improve baroreflex sensitivity at early stages of the disease and that HF diet reverses the beneficial effects of PARP-1 deletion on baroreflex function.

### PARP-1 Gene Deletion Improves HR Responses Following Selective Blockade of the Autonomic Nervous System

**Figure 3:**
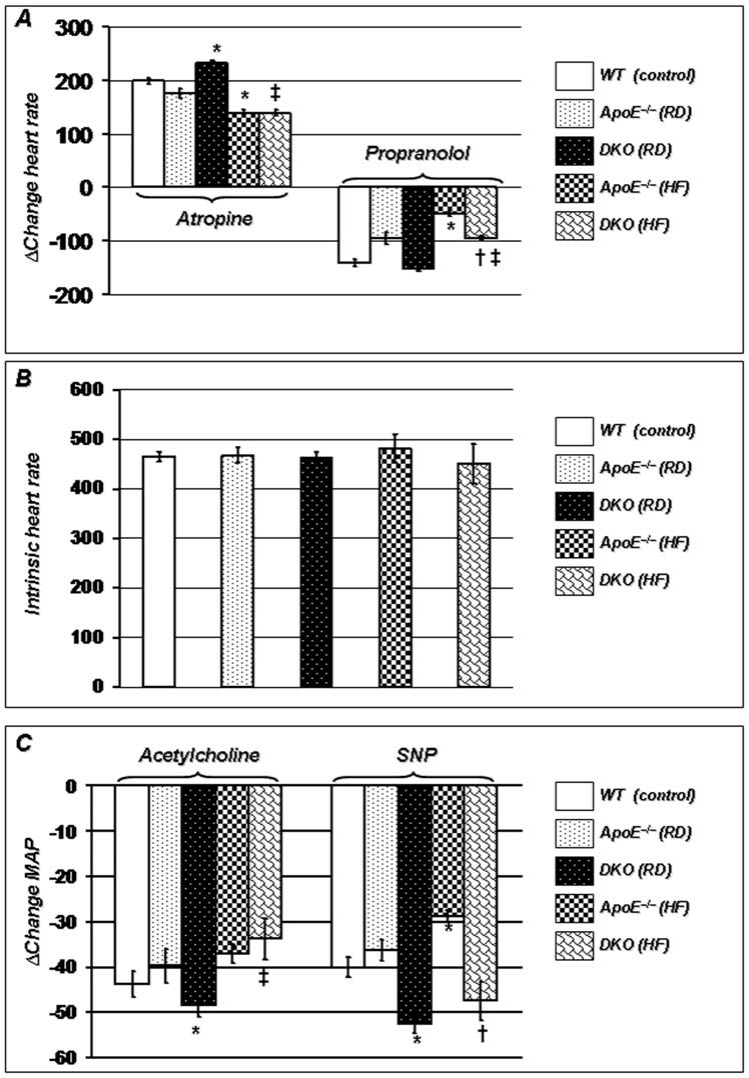
Loss of PARP-1 preserves autonomic and endothelial functions. (A). In response to atropine (parasympathetic tone blockade; 1 mg/kg), tachycardia was higher in DKO mice on RD than ApoE^−/−^, but high-fat diet abolished this effect. propranolol (sympathetic tone blockade; 8 mg/kg), induced higher bradycardic response in DKO than ApoE^−/−^ and high-fat diet did not abolish this effect, Intrinsic HR determined following hexamethonium treatment (ganglionic blockade; 20 mg/kg i.p.) was comparable among these groups (B). Increase in MAP after acetylcholine (endothelium dependent vasodilator; 2 mg/kg) in DKO mice was higher than ApoE^−/−^ mice (3C). In response to SNP, endothelium independent vasodilation was similar in ApoE^−/−^ and WT mice, it was improved in DKO mice. Interestingly, although HF diet further impaired the ability of the vessels to relax, endothelium independent vasodilation was totally preserved in DKO mice. *P* value (<0.05) *compared to ApoE^−/−^ RD, †compared to ApoE^−/−^ HF (16 weeks), and ‡compared to DKO mice on RD.

Blockade of the parasympathetic drive by atropine (1 mg/kg) resulted in similar tachycardic responses in both wild type (WT) and ApoE^−/−^ mice on RD, suggesting that vagal tone is not impaired in this group (Figure 3A). However, this increase in HR in response to atropine was significantly higher in DKO mice maintained on a regular diet than in ApoE^−/−^ mice on a similar diet regimen. HF diet similarly reduced parasympathetic tone in ApoE^−/−^ and DKO mice, again showing that the beneficial effect of PARP-1 gene deletion is reversed by HF diet. Blockade of sympathetic tone by propranolol (8 mg/kg), a non-specific β-blocker, resulted in higher bradycardic in WT mice compared to ApoE^−/−^ mice on RD suggesting that sympathetic tone is exacerbated in this mouse model of atherosclerosis (Figure 3B). This impairment was reversed in DKO mice fed RD. 16-week HF-diet regimen further exacerbated sympathetic tone in ApoE^−/−^ mice; DKO mice remain protected against increased sympathetic tone. To exclude the possibility that the beneficial effects of PARP-1 gene deletion on HR responses, following atropine or propranolol administration, were due to genetic alterations of cardiac function, a ganglionic blocker (hexamethonium; 20 mg/kg i.p.), was administered and intrinsic HR between the different groups was evaluated. As shown in Figure 3B, when complete autonomic outflow was attenuated, the intrinsic HR was comparable between the groups, showing that the alterations observed in response to atropine or propranolol were not due to changes in cardiac rhythm, but reflected significant alterations of autonomic function.

### PARP-1 Gene Deletion Prevents Dyslipidemia-Induced Endothelial Dysfunction

In order to establish the role of PARP-1 in endothelial dysfunction in conscious mice, the present study determined whether PARP-1 gene deletion provides protection against endothelium-dependent (acetylcholine; ACh) and independent (sodium nitroprusside; SNP) vasodilators. As shown in Figure 3C, both drugs lowered MAP in mice fed a RD. However, ACh-induced MAP reduction was significantly improved in DKO mice compared to ApoE^−/−^ mice, suggesting that endothelium-dependent vasodilation was preserved in these mice. After feeding a HF diet regimen for 16 weeks, both mouse strains exhibited similar impairments of endothelium-dependent vasodilation in response to ACh. Again, while endothelium independent vasodilation was similar in ApoE^−/−^ and WT mice on RD, it was significantly enhanced in DKO mice. Interestingly, although HF diet further impaired the ability of the vessels to relax, endothelium independent vasodilation was totally preserved in DKO mice. This further suggests that PARP-1 gene deletion is protective against endothelium-dependent dysfunction.

### PARP-1 gene deletion ameliorates the atherogenic index of high fat diet fed-ApoE^−/−^ mice

**Figure 4:**
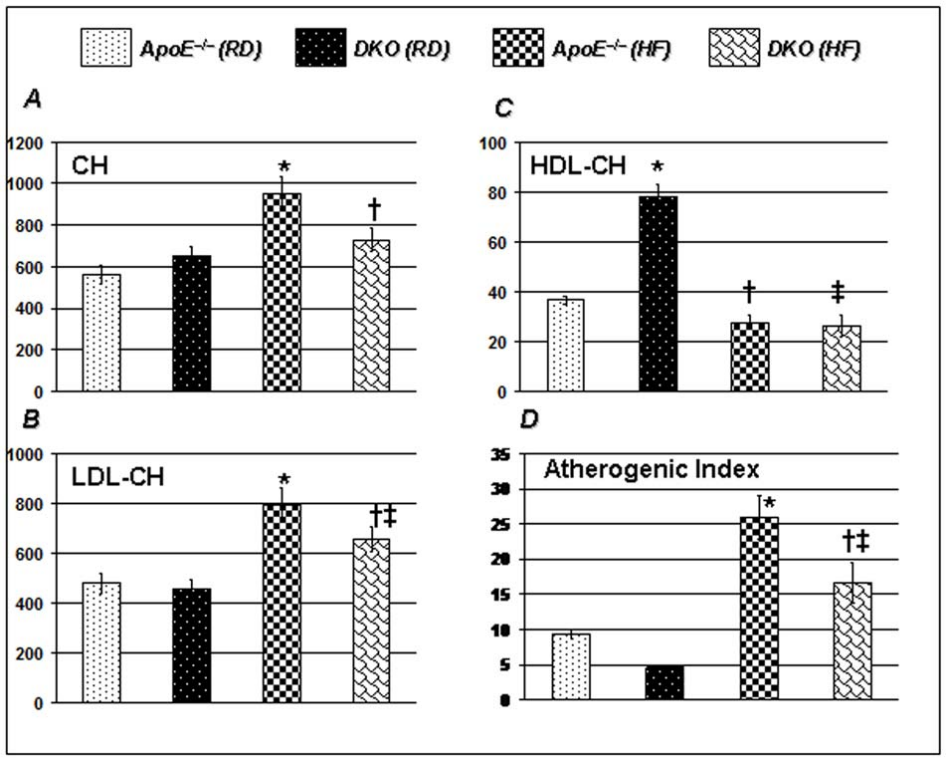
PARP-1 inhibition decreases atherogenic index in ApoE^−/−^ mice on HF diet for 16 weeks. Lipid profile data of 4 h starved mice shows that PARP-1 inhibition significantly increased HDL-cholesterol and total cholesterol in ApoE^−/−^ mice on regular diet (4A and 4C). After feeding HF diet for 16 weeks, total and LDL-cholesterol increased dramatically in ApoE^−/−^ mice. The increase in total and LDL cholesterol in DKO mice was significantly lower than ApoE^−/−^ mice on similar diet regimen (4A and 4B). Though HF diet significantly decreased HDL-cholesterol in DKO mice, atherogenic index still remained significantly higher in ApoE^−/−^ mice than DKO mice (Figure 2D). The data is expressed as mean±SEM from at least 6 mice in each group for 3 different set of experiments. *P* value (<0.05) *compared to age matched ApoE^−/−^ RD, †compared to ApoE^−/−^ HF (16 weeks), and ‡compared to age matched ApoE-PARP-1^d−/−^ mice on a regular diet.

Lipid profile data showed that DKO mice on RD exhibited moderately higher levels of LDL and total cholesterol (Figure 4). HDL-cholesterol in DKO mice on RD was markedly higher as compared to ApoE^−/−^ mice on similar diet regimen (Figure 4). As expected, a 16 weeks HF diet regimen dramatically increased total and LDL-cholesterol in ApoE^−/−^ mice. However, such an elevation in total and LDL-cholesterol was less pronounced in DKO mice. Atherogenic index is increasingly regarded as a more reasonable parameter of dyslipidemia rather than individual cholesterols [31]. In our study, the atherogenic index was significantly higher in ApoE^−/−^ than in DKO mice on RD as well as in response to HF diet for 16 weeks. These results suggest that dyslipidemia may be partly responsible for vascular and autonomic dysfunctions and that protective effects of PARP-1 inhibition on these abnormalities may be mediated partly through its effects on lipid profile (Figure 4).

### Loss of PARP-1 Preserves eNOS Activity without Altering Total eNOS Expression

**Figure 5:**
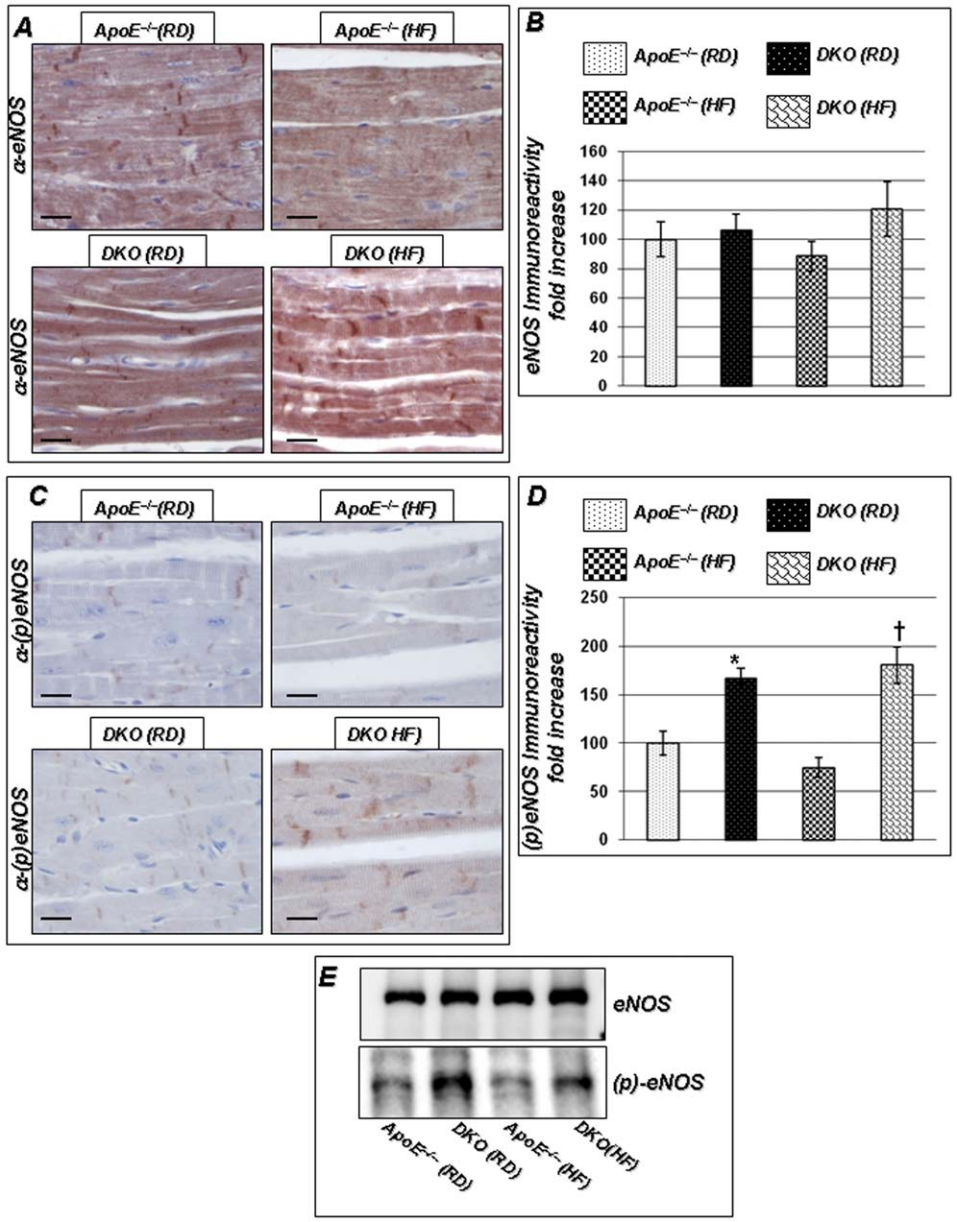
Lack of PARP-1-mediated protection against endothelium dysfunction is associated with increased eNOS activity. Figures 5A and C show IHC data with antibodies to eNOS or phospho-eNOS respectively of heart from ApoE^−/−^ and DKO mice on a regular or high-fat for 16 weeks. Figures 5 B and D represent quantitation of immunoreactivity, performed using Image-Pro Plus software and expressed as immunoreactivity/mm^2^. (E) Western Blot of left ventricles isolated from these experimental groups (n = 6) were collected and subjected to protein extraction and western blotted for phopho-eNOS, or total eNOS. Scale bar: 50 µm. *P* value (<0.05) *compared to ApoE^−/−^ RD, †compared to ApoE^−/−^ HF (16 weeks), and ‡compared to DKO mice on RD.

In ApoE^−/−^ mice, impaired responses to ACh could be due to an alteration in either eNOS expression or eNOS activation (eNOS phosphorylation). Immunohistochemical analysis of unmodified or phosphorylated eNOS in cardiac tissue isolated from the different experimental groups revealed that total eNOS expression was not altered when mice were maintained on a RD or HF diet (Figure 5A, B). However, phospho-eNOS immunoreactivity was significantly greater in DKO than in ApoE^−/−^ mice, following either regular or HF diet (Figure 5C, D). These observations were confirmed by immunoblot analysis of cardiac extracts using the same antibodies (Figure 5E) and by RT PCR on cDNA prepared from left ventricle of these mice (Figure S1).

### PARP-1 Gene Deletion Reduces Protein Nitration in Cardiac Tissue of HF-fed ApoE^−/−^ Mice

**Figure 6:**
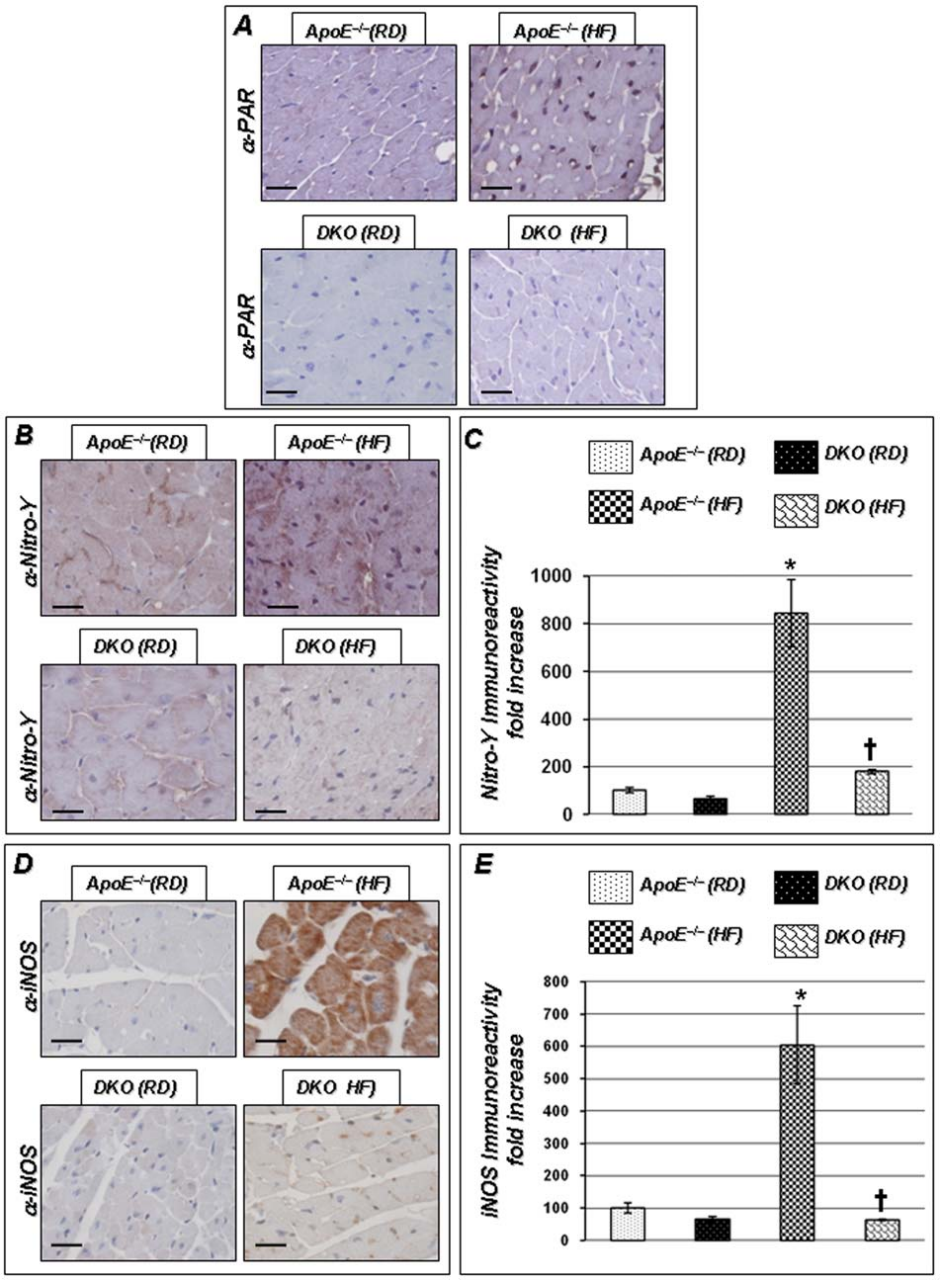
PARP-1 gene deletion-induced protection of endothelial dysfunction is associated with reduction in the expression of nitrotyrosine and iNOS in heart. Heart isolated from these experimental groups were collected and subjected to immunohistochemistry with antibodies to PAR (A), nitrotyrosine (B) and iNOS (C). Figures 6C and E represent quantitation of immunoreactivity was conducted using Image-Pro Plus software and expressed as immunoreactivity/mm^2^. Scale bar: 50 µm. *P* value (<0.05) *compared to ApoE^−/−^ RD, †compared to ApoE^−/−^ HF (16 weeks), and ‡compared to DKO mice on RD.

Cardiac tissue from the HF diet-fed ApoE^−/−^ mice exhibited a high poly-ADP-ribose (PAR) immunoreactivity, indicating that PARP-1 activity was elevated in these mice, while a significantly lower PAR immunoreactivity was detected in ApoE^−/−^ mice receiving a RD (Figure 6A). No PAR immunoreactivity was detected in cardiac tissue prepared from HF or RD-fed DKO mice. PARP-1 activation was mirrored by an increase in protein nitration as assessed by nitro-tyrosine immunoreactivity (Figures 6B&C), establishing a potential association between PARP-1 activation and oxidative stress. Protein nitration was decreased in tissue of HF diet-fed DKO mice (Figures 6D&E).

## Discussion

The results of the present investigation demonstrate that PARP-1 participates in dyslipidemia-mediated vascular dysfunction and dysregulation of autonomic function and that PARP-1 gene deletion is protective against these defects. Previously, our laboratory demonstrated that PARP-1 inhibition affects atherosclerotic plaque dynamics and these alterations are highly desirable for the control of plaque progression and promotion of stability [15], [16]. Dynamics of atherogenesis are confined to atherosclerotic plaques but are integral participants of overall vascular dysfunction including hypertension, myocardial infarction and heart failure. The present study is a direct continuation of our ongoing investigation of the role of PARP-1 in atherosclerosis and associated pathologies.

Alterations in central nervous system control of BP and HR are primary outcomes of dyslipidemia. Thus, devising strategies that can diminish or completely block the manifestation of these outcomes is of great clinical importance. In this study, we demonstrate that PARP-1 plays an important role in both dyslipidemia-associated vascular and autonomic dysfunction. Inhibition of this enzyme by gene knockout partially improved baroreflex sensitivity, without affecting baseline hemodynamic parameters, and improved autonomic function. It also prevented dyslipidemia-induced endothelial dysfunction, possibly through maintenance of eNOS activity. Moreover, modulation of oxidative tissue damage may underlie the observed protective effects of PARP-1 gene knockout.

The arterial baroreceptor reflex is a classic negative feedback mechanism that is critical for maintaining BP around a setpoint by sensing its fluctuations and correcting accordingly [32], [33]. In recent years, a great deal of evidence has accumulated that supports the ability of dyslipidemia and its associated pathologies to impair SBRS in humans [32], [33]. In fact, Nasr et al. recently showed that bilateral carotid atherosclerosis in human patients is associated with an impairment of SBRS and a shift of the sympathovagal balance toward a decrease in parasympathetic-associated HR variability[33]. Many of these changes are clearly associated with elevated oxidative stress. Our laboratory previously reported that oxidative stress contributes to autonomic dysregulation in hypertensive and hypercholesterolemic mice[30]. The negative effect of dyslipidemia on SBRS observed is consistent with our previous studies done in ApoE^−/−^ mice, with the effects of dyslipidemia observed in humans as well as in LDL-receptor knockout mice, as very recently reported by Campos et al[34]. It is noteworthy that changes in SBRS regulation may be mainly mediated by parasympathetic tone, an idea also set forth by Tank et al[35]. Furthermore, the parasympathetic tone seems more sensitive to dyslipidemia than sympathetic tone. A rather important and interesting finding in this study is that PARP-1 gene deletion preserved SBRS in regular diet-fed mice, but this protective effect was completely abolished in HF diet-fed animals. The mechanism through which a HF diet overwhelms the protective effect of PARP-1 gene deletion is unknown.

PARP-1 gene deletion preserved parasympathetic tone in RD-fed mice, but failed to do so in mice fed a HF diet. Conversely, PARP-1 deficiency significantly prevented exacerbated sympathetic tone not only in regular diet-fed ApoE^−/−^ mice, but also in HF diet-fed animals. The reason(s) for the ability of DKO mice to maintain these responses during a regular diet regimen is unclear, and the reason(s) for their ability to maintain parasympathetic tone responses during a high-fat diet is even more puzzling. These discrepancies are not unprecedented, as Rose et al[36] reported that PI3K^−/−^ mice displayed similar responses, which were attributed to negative regulation of cAMP by PI3K in the ventricular myocardium. Whether PARP-1 deficiency may affect the autonomic nervous system through a mechanism involving PI3K activity and cAMP is unknown, although poly(ADP-ribosyl)ation of the PI3K family member, DNA-dependent protein kinase, has been shown to enhance its kinase activity [37]. However, as eluded to earlier, oxidative stress has become increasingly regarded as an important participant in vascular impairments. Therefore, PARP-1 gene deletion may preserve cardiovascular function by modulating factors that instate oxidative stress. Inhibition of iNOS overexpression and subsequent nitrergic damage may be one such example.

Our data suggests that though PARP-1 gene deletion moderately increased total cholesterol levels on regular diet regimen, but HF diet induced higher total and LDL- cholesterol in ApoE^−/−^ mice than DKO mice. Moreover, PARP-1 gene deletion significantly increased HDL-cholesterol, an effect which was abolished by HF diet. Interestingly, von Lukowicz et al[18] reported significantly higher levels of total and LDL- cholesterol in DKO mice as compared to ApoE^−/−^ mice which is in contrast to our data. However, in their study, they used 1.25% total cholesterol in their diet which is 6 times higher than HF diet used in our studies (0.2% cholesterol). Further mice used in our study are from homogenous background (C57BL/6J). Atherogenic index, which has been suggested as better index of dyslipidemia and important tool to analyze the results considering the complexity of lipoproteins[31] was significantly decreased by PARP-1 gene deletion in both RD and HF regimen. Interestingly PARP-1 gene deletion increased HDL by double in regular diet group, an effect which was abolished by HF diet. Though mechanism of increased HDL levels by PARP-1 gene deletion is unknown, HDL- cholesterol has independently been reported to correlate with autonomic and vascular dysfunctions[38], [39]. *ApoE^−/−^ mice can develop atherosclerotic lesions while on a regular diet but require a lengthy duration* [*40*] *. In fact, complicated and multilayered plaques lesions such as those observed in humans require near or more than a year to take place. However, it remains rather interesting and important to assess the effect of long term regular diet on the deterioration of autonomic and vascular functions and protective effects by PARP inhibition.*

Many studies, including those from our laboratory, have shown that PARP-1 inhibition greatly reduces expression of iNOS[41], [42]. Several groups, including ours, have demonstrated the responsiveness of PARP-1 to oxidative stress and its participation in oxidative stress-associated endothelial and cardiac dysfunctions [15], [16], [19]. In fact, our laboratory recently demonstrated that a more complex relationship between PARP-1 and iNOS exists, as these two proteins are reciprocally regulated during airway inflammation with NF-κB activation serving as major component of this relationship[43]. Notably, NF-κB regulates the expression of iNOS during inflammation[42]. NF-κB has also been suggested to regulate NADPH oxidase subunit p22 (phox) in human aortic smooth muscle cells[44]. Therefore, PARP-1 deficiency may exert an antioxidant effect and maintain autonomic nervous system through a direct effect on NAD(P)H oxidase complex function. Obviously, this hypothesis needs to be confirmed through additional experimentation.

Endothelial NOS (eNOS) activity is thought to have an important influence on the activity of the sympathetic tone[45]. Pathological processes that reduce vascular NO may do so through down regulation of eNOS expression or inhibition of eNOS activity. They may also reduce vascular NO by decreasing NO bioavailability, an effect attributable to NO inactivation by radicals such as O_2_^−^, which generates the noxious byproduct, peroxynitrite (ONOO^−^)[46]. Thus, increased oxidative stress in the vascular wall is a key mechanism underlying endothelial dysfunction[46]. The increased levels of protein nitration in cardiac tissues of HF diet-fed animals is evidence for increased levels of ONOO−, which would be suggestive of a decrease in NO bioavailability and may explain the observed endothelial dysfunction in these animals[47]. Therefore, it is tempting to speculate that the ability of PARP-1 deficiency to prevent such extensive oxidative stress and to maintain eNOS activity underlies protection against endothelial dysfunction and dysregulated autonomic nervous system.

Numerous studies of PARP-1 involvement in oxidative stress-associated endothelial dysfunction, primarily using cell culture and *ex vivo* systems, have demonstrated that pharmacological inhibition of PARP-1 confers protection against such dysfunction (for review[19], [23]).

In conclusion, using conscious animals we have demonstrated that PARP-1 participates in dyslipidemia-mediated vascular dysfunction and dysregulation of autonomic function and that PARP-1 gene deletion is protective against these defects. Future studies may better clarify the mechanism by which PARP-1 participates in autonomic nervous system function.

## Supporting Information

Figure S1 RT PCR shows similar eNOS expression in heart or aorta of ApoE−/− mice and DKO mice suggesting that PARP-1 inhibition is affecting activation (phosphor-eNOS) rather than amount of eNOS. cDNA generated from the heart or aorta extract was subjected to PCR using the primers described in the Materials and Methods generating a single PCR product.
(0.06 MB TIF)
