# Supplementary figures and images for: Protective Effects of PARP-1 Knockout on Dyslipidemia-Induced Autonomic and Vascular Dysfunction in ApoE−/− Mice: Effects on eNOS and Oxidative Stress
Source: PLoS One. 2009 Oct 13;4(10):e7430. doi: 10.1371/journal.pone.0007430 (PMC2757717; doi:10.1371/journal.pone.0007430)

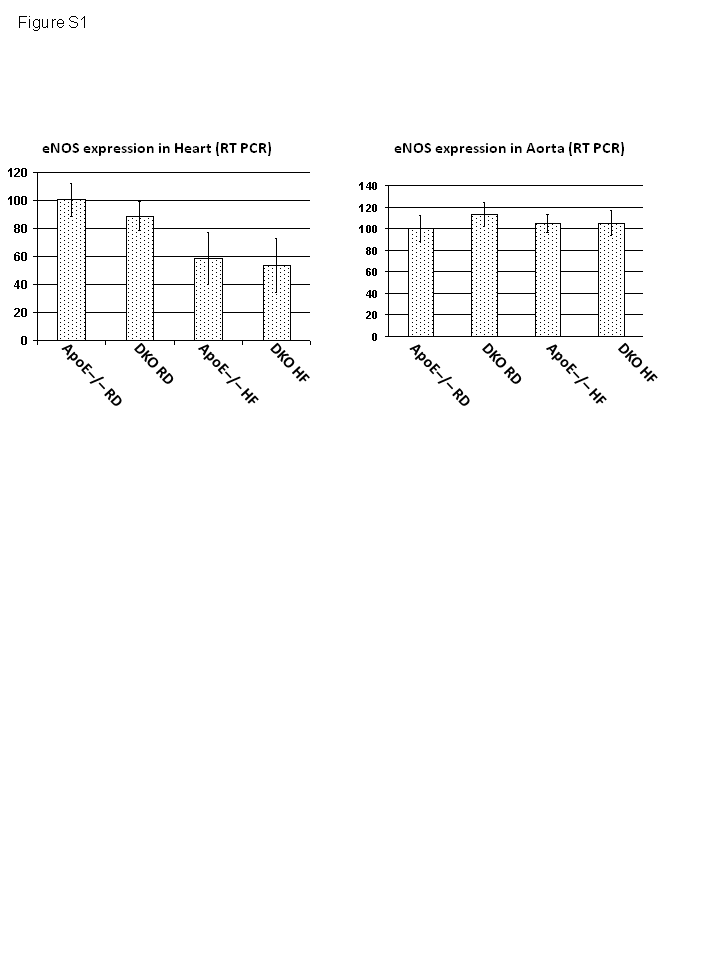

Supplement: Figure S1 — RT PCR shows similar eNOS expression in heart or aorta of ApoE−/− mice and DKO mice suggesting that PARP-1 inhibition is affecting activation (phosphor-eNOS) rather than amount of eNOS. cDNA generated from the heart or aorta extract was subjected to PCR using the primers described in the Materials and Methods generating a single PCR product. (0.06 MB TIF) [file pone.0007430.s001.tif]
